# Blood Purification in Patients with Sepsis Associated with Acute Kidney Injury: A Narrative Review

**DOI:** 10.3390/jcm12196388

**Published:** 2023-10-06

**Authors:** Jun Kamei, Masafumi Kanamoto, Yutaka Igarashi, Kodai Suzuki, Kensuke Fujita, Tsukasa Kuwana, Takayuki Ogura, Katsunori Mochizuki, Yuki Banshotani, Hiroyasu Ishikura, Yoshihiko Nakamura, J-STAD (Japan Sepsis Treatment and Diagnosis) Study Group

**Affiliations:** 1 Department of Primary Care and Emergency Medicine, Graduate School of Medicine, Kyoto University, Kyoto 606-8501, Japan; junk19860816@gmail.com; 2 Department of Anesthesiology, Gunma Prefectural Cardiovascular Center, 3-12, Kameizumi, Gunma 371-0004, Japan; kanamoto@gunma-u.ac.jp; 3 Department of Emergency and Critical Care Medicine, Nippon Medical School, Tokyo 1138603, Japan; igarashiy@nms.ac.jp; 4 Department of Emergency and Disaster Medicine, Gifu University Graduate School of Medicine, Gifu 501-1194, Japan; kodaisuzuki@ymail.ne.jp; 5 Department of Emergency Medicine and Critical Care Medicine, Saiseikai Utsunomiya Hospital, Tochigi 321-0974, Japan; fujitak.727@gmail.com (K.F.); alongthelongestway2003@yahoo.co.jp (T.O.); 6 Division of Emergency and Critical Care Medicine, Department of Acute Medicine, Nihon University School of Medicine, Tokyo 173-8610, Japan; kuwana.tsukasa@nihon-u.ac.jp; 7 Department of Emergency and Critical Care Medicine, Osaka Medical and Pharmaceutical University, Osaka 569-8686, Japan; katsunori.mochizuki@ompu.ac.jp; 8 Tajima Emergency & Critical Care Medical Center, Toyooka Hospital, Hyogo 668-8501, Japan; ybanchan33@yahoo.co.jp; 9 Department of Emergency and Critical Care Medicine, Fukuoka University Hospital, Fukuoka 814-0180, Japan; ishikurah@fukuoka-u.ac.jp

**Keywords:** sepsis, acute kidney injury, kidney replacement therapy, renal indication, non-renal indication

## Abstract

Sepsis leads to organ dysfunction. Acute kidney injury, a common type of organ dysfunction, is associated with a high mortality rate in patients with sepsis. Kidney replacement therapy can correct the metabolic, electrolyte, and fluid imbalances caused by acute kidney injury. While this therapy can improve outcomes, evidence of its beneficial effects is lacking. Herein, we review the indications for blood purification therapy, including kidney replacement therapy, and the current knowledge regarding acute kidney injury in terms of renal and non-renal indications. While renal indications have been well-documented, indications for blood purification therapy in sepsis (non-renal indications) remain controversial. Excessive inflammation is an important factor in the development of sepsis; blood purification therapy has been shown to reduce inflammatory mediators and improve hemodynamic instability. Given the pathophysiology of sepsis, blood purification therapy may decrease mortality rates in these patients. Further trials are needed in order to establish the effectiveness of blood purification therapy for sepsis.

## 1. Introduction

Sepsis is defined as life-threatening organ dysfunction caused by a dysregulated host response to infection [1]. Acute kidney injury (AKI) is a common type of organ dysfunction and is associated with a high mortality rate in patients with sepsis [2]. Kidney replacement therapy (KRT) can correct the metabolic, electrolyte, and fluid imbalances caused by severe AKI. Current research is focused on the optimal method of KRT for septic AKI, especially regarding the timing of initiation [3].

In addition to maintaining homeostasis (renal indication), blood purification therapy (BPT), including KRT, regulates excessive inflammatory responses (non-renal indication). In non-renal indications, BPT is used to reduce the levels of pathogenic substances and as an adjunctive therapy for sepsis; however, while it can improve patient outcomes, evidence on its beneficial effects is lacking [4,5].

Therefore, in this study, we aimed to review the indications for BPT and the current knowledge regarding AKI. We also summarized the current evidence for the renal and non-renal indications for BPT and present future directions for study.

## 2. Indications for Acute BPT in Sepsis

Acute BPT is performed for renal function support during AKI (renal indication) and to remove or supply various substances related to pathological conditions (non-renal indication). Patients with sepsis receive acute BPT for both renal and non-renal indications.

### 2.1. Renal Indications

Approximately 50% of the sepsis cases are complicated by AKI [6,7,8], and septic AKI is strongly associated with mortality and long-term hospitalization [9]. Moreover, severe AKI, characterized by the progression of metabolic acidosis, hyperkalemia, uremia, oliguria/anuria, and excess fluid volume, is life-threatening. Therefore, KRT for renal indications, such as intermittent hemodialysis and continuous hemofiltration, is performed to remove waste products that are otherwise processed by the kidneys and manage fluid volumes and electrolytes.

### 2.2. Non-Renal Indications

In contrast, BPT for non-renal indications in sepsis cannot be used as a substitute for renal function. Instead, it is used to improve survival outcomes by removing pathogenic and pathogen-related substances that cause sepsis and cannot be removed via normal renal function. In patients with sepsis, inflammatory and anti-inflammatory mediator levels are positively correlated with mortality [6,10]. The pathological conditions are expected to improve upon the removal or adsorption of endogenous mediators such as alarmins, represented by high mobility group box protein 1 [6]. Continuous hemofiltration and hemodiafiltration occurs as BPTs remove cytokines and reduce their concentration in the blood and tissue [11]. Specifically, in the peak concentration hypothesis, the maximum concentration of cytokines in the blood is reduced by removing cytokines with continuous KRT (CKRT) [12]. In addition, according to the threshold modulating hypothesis, reducing the concentration of cytokines in tissues hinders further production of local cytokines, thus leading to the reduction of the overall cytokine concentrations [13]. BPT also increases the lymphatic flow rate, which allows cytokines in tissues to be removed (mediator delivery hypothesis) [14]. Furthermore, blood adsorption therapy (hemoadsorption) may be performed to remove toxic and pathogenic substances such as endotoxins and mediators from the blood with an adsorbent.

## 3. Definition and Staging Criteria of AKI

Different diagnostic criteria have been used for acute renal failure in more than 30 countries (Table 1); however, the Acute Dialysis Quality Initiative published the risk, injury, failure, loss, and end-stage renal disease (RIFLE) criteria in 2004 [15]. The RIFLE criteria consist of an increase in serum creatinine level, a decrease in the glomerular filtration rate, and a decrease in urine output. Later, minor elevations in serum creatinine levels were found to affect the clinical course and outcome, and the Acute Kidney Injury Network (AKIN) proposed the concept of AKI, which included early stages of kidney injury. In 2007, the AKIN criteria, a modified version of the RIFLE, were published, adding a mildly elevated creatinine concentration (0.3 mg/dL) and time course (within 48 h) as diagnostic criteria [16]. The Kidney Disease Improving Global Outcomes (KDIGO) organization published guidelines for AKI and proposed the KDIGO criteria in 2012, which integrated the RIFLE and AKIN criteria [17]. The KDIGO criteria include patients exhibiting gradual deterioration and have been reported to be superior to the RIFLE and AKIN criteria in predicting outcomes [18,19,20]. All diagnostic criteria include a broad spectrum of disease.

## 4. Biomarkers for AKI

As acute tubular necrosis is preceded by an increase in serum creatinine levels, an early diagnosis of acute tubular necrosis cannot be achieved, which may delay the treatment. However, several candidates have been identified as sensitive biomarkers for AKI, including urinary tubular enzymes (e.g., proximal renal tubular epithelial antigen, alpha-glutathione S-transferase, pi-glutathione S-transferase, gamma-glutamyltranspeptidase, alanine aminopeptidase, lactate dehydrogenase, N-acetyl-beta-glucosaminidase, and alkaline phosphatase), urinary low-molecular-weight proteins (e.g., alpha-1-microglobulin, beta-2-microglobulin, retinol-binding protein, adenosine deaminase-binding protein, and cystatin C), neutrophil gelatinase-associated lipocalin (NGAL), urinary kidney injury molecule-1, urinary interleukin (IL)-18, and L-type fatty acid-binding protein (L-FABP). Urinary NGAL and L-FABP have been suggested to be useful early diagnostic markers of AKI. NGAL represents an early biomarker of ischemic renal injury and is markedly upregulated in children undergoing cardiac surgery [21]. Moreover, NGAL levels are elevated 1–3 days before the increase in serum creatinine levels [22]. L-FABP is located in the proximal tubules and correlates with the severity of ischemic tubular damage [23], demonstrating that it can identify AKI and predict in-hospital mortality and the need for hemodialysis [24]. Only NGAL and TIMP-2·IGFBP7 (Nephrocheck™, bioMérieux, Durham, NC, USA), the product of the tissue inhibitor of metalloproteinase 2 and insulin-like growth factor-binding protein 7, are clinically used. TIMP-2·IGFBP7 is a urinary marker of cell cycle arrest, reflecting cellular stress that precedes tissue damage. It is approved by the Food and Drug Administration and European Medicines Evaluation Agency for the prediction of AKI stage 2 and 3 within 12 h in critically-ill patients with cardiac and respiratory failures [25].

## 5. Epidemiology and Pathophysiology of Septic AKI

To diagnose sepsis-induced AKI, the independent criteria for both sepsis and AKI described above must be met. In 2016, sepsis was redefined as serious organ dysfunction due to a dysregulated host response to infection [1]. This definition focuses on more severe septic cases than those included in the previous criteria associated with systemic inflammatory response syndrome [26]. Sepsis occurs in approximately 80% of AKI cases [27]. The outcomes of septic AKI have improved in recent years due to KRT [28]. However, the 60-day hospital mortality rate of patients with septic shock who develop stage 2-to-3 AKI is approximately 5-fold greater than that of those without AKI [29]. Thus, novel epidemiological studies are required in order to evaluate sepsis-induced AKI under its new definition.

The pathophysiology of AKI in sepsis is complex and remains largely unknown. Decreased renal blood flow (RBF) resulting from several factors such as hypovolemia, hypotension, and septic cardiomyopathy is the main cause of ischemic AKI, including sepsis-induced AKI [30]. However, patients with septic AKI do not always have low RBF. After endotoxin administration, RBF reportedly declined in some studies using mouse models and increase in others [31]. In either case, the model mice developed AKI.

**Figure 1:**
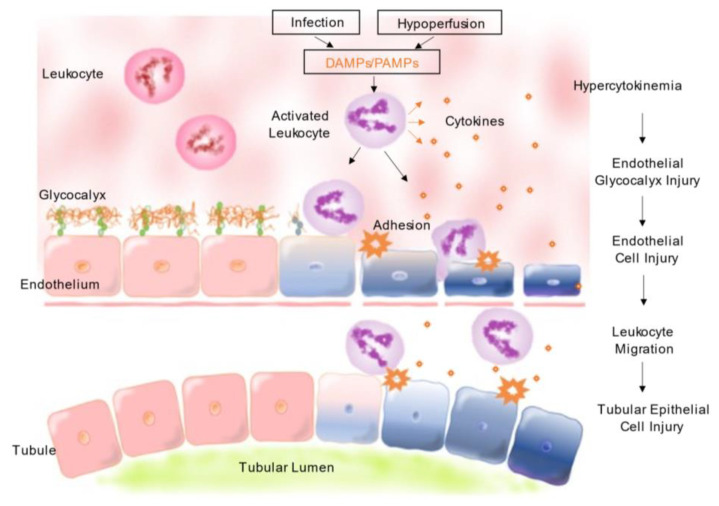
Pathophysiological mechanism of septic acute kidney injury. DAMPs/PAMPs promote activation of circulating leukocytes, which then release cytokines, such as TNFα, IL-1, IL-6, and IL-8. Hypercytokinemia damages the vascular endothelial glycocalyx and vascular endothelial cells. Endothelial glycocalyx shedding promotes leukocyte rolling, adhesion, and migration in the interstitial space, which results in tubular epithelial cell injury. DAMPs: damage-associated molecular patterns; PAMPs: pathogen-associated molecular patterns; TNF: tumor necrosis factor; IL: interleukin.

Septic AKI differs from other types of AKI due to massive release of inflammatory mediators, exogenous pathogen-associated molecular patterns (PAMPs), and endogenous damage-associated molecular patterns (DAMPs) during bacterial, fungal, or viral infection [32]. Endotoxins, found in the outer membrane of gram-negative bacteria, are a type of PAMP that activate the leukocytes and toll-like receptor family, leading to the activation of various intracellular signaling pathways via nuclear factor-κB [33], inducing the release of inflammatory cytokines tumor necrosis factor (TNF)-α, IL-1, IL-6, and IL-8 [34]. This response sometimes results in hyperinflammation, which can lead to vascular endothelial damage. Injury of the endothelial glycocalyx, which covers the surface of vascular endothelial cells, is an essential component of vascular endothelial damage. Moreover, the endothelial glycocalyx is involved in microvascular homeostasis and maintains endothelial permeability and microvascular tone, preserves an oncotic gradient across the endothelial barrier, and modulates adhesion and migration of leukocytes [35]. Injected endotoxins degrade the endothelial glycocalyx of glomerular capillaries in the kidneys [36], resulting in septic AKI without hypoperfusion of RBF. Moreover, renal tubular epithelial cells (TECs) express toll-like receptors and PAMPs/DAMPs in the glomerulus, increase oxidative stress, and lead to the production of reactive oxygen species as well as mitochondrial injury [37]. The pathophysiological mechanism of septic AKI is shown in Figure 1.

## 6. KRT Initiation Timing for AKI

Presently, the timing of CKRT initiation for AKI has not been established. The Surviving Sepsis Campaign Guidelines (SSCG) 2021 [38] recommend not to introduce KRT in AKI patients without absolute renal indications among those with sepsis or septic shock (Table 2). These guidelines remain unchanged from the SSCG 2016. Conversely, in the Japanese Clinical Practice Guidelines for Management of Sepsis and Septic Shock 2020 (J-SSCG 2020) [39], the degree of recommendation is based on the disease stage. The J-SSCG 2020 does not recommend KRT for stage 2 vs. stage 3 or classical absolute adaptations and suggests “against initiating KRT at stage 3 for patients with septic AKI rather than absolute indications.” Although multiple randomized controlled trials (RCTs) have compared the prognosis between early and late initiation of KRT, the definitions of “early” and “late” vary. Table 1 shows a comparison of the early versus late initiation of renal replacement therapy in critically ill patients with acute kidney injury (ELAIN) [40], the artificial kidney initiation in kidney injury (AKIKI) [41], initiation of dialysis early versus delayed in the intensive care unit (IDEAL-ICU) [42], standard versus accelerated initiation of renal replacement therapy in acute kidney injury (STARRT-AKI) [43], and AKIKI-2 criteria [44]. In addition, the patient background (percentage of postoperative patients or patients with sepsis), inclusion of patients with pulmonary edema, and CKRT rates differ among studies. In the ELAIN trial, the early initiation of KRT in patients with stage 2 AKI as per the KDIGO criteria and a plasma NGAL level > 150 ng/mL improved mortality, whereas in other studies it did not. The AKIKI-2 study [44], which investigated delayed initiation of KRT at oliguria or a blood urea nitrogen (BUN) concentration ≥ 112 mg/dL for ≥ 72 h, as well as further delayed initiations at absolute indications or a BUN concentration ≥ 140 mg/dL, showed that the further delayed initiation strategy increased the risk of death.

AKI is an independent risk factor for sepsis-related mortality [2]. Although the KDIGO clinical practice guidelines recommend early initiation of KRT in patients with potentially life-threatening changes in fluid overload, acid-base balance, or electrolyte imbalance (no grade) [17], the corresponding evidence is lacking. Moreover, the mechanism and pathophysiology of sepsis-induced AKI are significantly different from those of other types of AKI. Therefore, the timing of KRT initiation should be based on patient-specific characteristics.

## 7. Intensity of KRT

In the past several decades, several clinical trials have focused on optimizing the intensity of CKRT. For example, in 2000, Ronco et al. [45] reported on patients with AKI admitted to the intensive care unit and randomly subjected to ultrafiltration at 20, 35, and 45 mL/kg/h. They observed that the survival rate at 15 days in the 35 or 45 mL/kg/h groups was significantly higher than that in the 20 mL/kg/h group (*p* = 0.0007 and *p* = 0.0013, respectively). However, in 2008, Palevsky et al. [46] (VA/NIH, Acute Renal Failure Trial Network) reported an RCT in which critically-ill patients with AKI requiring KRT were randomly assigned to a 35 mL/kg/h group (intensive treatment strategy group) or a 20 mL/kg/h group (less-intensive treatment strategy group). The intensive treatment strategy group did not exhibit a lower mortality rate than the less-intensive RCT group (odds ratio [OR], 1.09; 95% confidence interval [CI], 0.86–1.40; *p* = 0.47). Similarly, in 2009, Bellomo et al. (Renal Replacement Therapy Study Investigators) [47] reported that in a large-scale RCT on critically-ill patients with AKI, the higher-intensity continuous KRT group (40 mL/kg/h) did not experience a reduced mortality rate at 90 days compared with that in the lower-intensity therapy group (25 mL/kg/h) (OR, 1.00; 95% CI, 0.81–1.23; *p* = 0.99). Finally, a systematic review and meta-analysis [48] showed that high-dose CKRT did not reduce mortality at 28 days (risk ratio, 0.88; 95% CI, 0.70–1.11; *p* = 0.28) in critically-ill patients with AKI. Overall, these findings suggest that high-intensity KRT is less beneficial in critically ill patients with AKI.

## 8. AN69/AN69ST/oXiris

The AN69 membrane is a copolymer of acrylonitrile and sodium methallyl sulfonate and a cytokine-adsorbing hemofilter [49]. The combined use of AN69 membrane and angiotensin-converting enzyme inhibitor during hemodialysis leads to elevated levels of bradykinin, indicating activation of the kallikrein–kinin system [50]. The newly developed AN69ST is an AN69 membrane that has been surface-treated with polyethyleneimine [49]. This modification leads to decreased kinin generation in the plasma and dialysate triggered by kininogen binding and activation at the AN69ST membrane compared to that observed with AN69 [50]. The oXiris is an AN69-based membrane with a positively charged polyethyleneimine layer capable of adsorbing negatively-charged endotoxin molecules [51]. These hemofilters may contribute to improved survival rates in patients with sepsis. The AN69 was evaluated in several RCTs in patients with septic shock and/or multiple organ failure [52,53,54], sepsis [55], and severe burns with bacteremia [56]. The levels of several mediators, including cytokines, such as TNF-α, IL-1β, IL-4, IL-6, IL-8, IL-10, and IL-13, and endotoxins, were significantly lower in the AN69 group than in the control group [53,54,55,56]. However, none of the RCTs have evaluated its impact on mortality or the effect of AN69ST on patients with sepsis. Furthermore, a previous RCT [57] showed that endotoxin levels decreased in seven out of the nine (77.8%) patients in the oXiris group and in only one out of the six (16.7%) patients in the standard filter group (*p* = 0.02). Thus, cytokine levels decreased more when the oXiris filter was used than when the standard filter was used. However, whether oXiris can reduce the mortality rate remains unclear.

## 9. Polymethyl Methacrylate

Polymethyl methacrylate (PMMA) is a unique membrane material that can absorb various inflammatory cytokines [58]. In an in vitro study, more than half of the 48 cytokines were absorbed and reduced by more than 50%, proving PMMA to be superior to polysulfone. Cytokines with molecular weights between 10,000 and 30,000 Da were absorbed with high efficiency, while those with molecular weights greater than 30,000 Da tended to be absorbed at a lower rate [59]. Few studies have demonstrated the clinical utility of PMMA membranes [60,61,62]; however, none of the RCTs have evaluated the incurred survival benefits. A single-center, retrospective study showed that sepsis patients treated with PMMA had a significantly lower 28-day survival rate than those treated with AN69ST (50.0% vs. 77.3%, *p* < 0.05) [63]. In contrast, the 28-day mortality rate was not significantly different between the two groups (43.8% in the AN69ST group vs. 35.3% in the PMMA group, *p* = 0.1625) [64].

## 10. CytoSorb^®^

CytoSorb^®^ (Cytosorbents, Corporation, Princeton City, NJ, USA) is a hemoadsorption device, which contains porous polymeric beads capable of removing cytokines and toxins from the blood. The first RCT to evaluate the effect of CytoSorb^®^ on patients with sepsis was conducted by Schadler et al. [65], wherein patients were randomly assigned to receive either CytoSorb^®^ or no hemoperfusion. The primary outcome of the study was the change in normalized IL-6 serum concentrations; no significant difference was observed between the two groups in IL-6 concentration (*p* = 0.15) or mortality (*p* = 0.19) after baseline normalization. In an RCT by Hawchar et al. [66], 20 patients with septic shock were randomized into CytoSorb^®^ (*n* = 10) and control groups (*n* = 10). In the CytoSorb^®^ group, the required dose of norepinephrine (*p* = 0.016) and procalcitonin concentration (*p* = 0.004) were significantly lower. However, the sample size in this RCT was too small. Recently, the Cytokine hemoadsorption during cardiac surgery versus standard surgical care for infective endocarditis (REMOVE) study, [67], a multicenter RCT, evaluated patients with infective endocarditis undergoing cardiac surgery. The primary outcome was the change in the sequential organ failure assessment (SOFA) score, and the secondary outcomes included the 30-day mortality rate, duration of mechanical ventilation, vasopressor therapy, and KRT. A total of 288 patients were randomly assigned to the CytoSorb^®^ (*n* = 142) or control (*n* = 146) groups. However, the change in SOFA score (*p* = 0.677), mortality at 30 days (21% CytoSorb^®^ vs. 22% control, *p* = 0.782), duration of mechanical ventilation (*p* = 0.165), vasopressor therapy (*p* = 0.896), and KRT (*p* = 0.791) did not differ between the two groups. Thus, previous RCTs have not shown clinical benefits of CytoSorb^®^ therapy for patients with sepsis. Alongside an ongoing multicenter RCT, PROCYSS, whose results are awaited, further evaluation is needed for a more conclusive perspective (NCT04963920).

## 11. Polimyxin B-Immobilized Fiber Column Direct Hemoperfusion

Polimyxin B-immobilized fiber column direct hemoperfusion (PMX-DHP; Toraymyxin^®,^ Toray Medical Co., Ltd. Tokyo, Japan.) is a type of BPT used to treat peritonitis and septic shock through endotoxin adsorption. Three RCTs have evaluated the effects of PMX-DHP. The first was the early use of polymyxin B hemoperfusion in abdominal septic shock (EUPHAS) trial [68], conducted in Italy in 2009 for abdominal septic shock. PMX-DHP, in addition to conventional therapy, significantly improved hemodynamics and organ dysfunction and reduced 28-day mortality rates (32.4% in the PMX-DHP group vs. 53.3% in the conventional group, *p* = 0.13). However, the 28-day mortality rate was not the primary endpoint, and the number of cases was small (11/34 in the PMX-DHP group vs. 16/30 in the conventional therapy group) [69]. The second trial, the ABDO-MIX trial [70] for abdominal septic shock, was conducted in France in 2015 and showed no significant difference between the PMX-DHP and conventional groups regarding the 28-day mortality (27.7% in the PMX-DHP group vs. 19.5% in the conventional group, *p* = 0.14). However, this study reported dramatically higher cartridge clotting and failure rates than those in other trials or clinical experience to date, suggesting technical issues in the implementation of the therapy protocol [71]. In response to these results, the SSCG 2016 stated [72]: “we make no recommendation regarding the use of blood purification techniques.” Additionally, the EUPHAS and ABDO-MIX trials did not measure blood endotoxin levels. The third trial, namely, the evaluating the use of polymyxin B hemoperfusion in an RCT of adults treated for endotoxemia and septic shock (EUPHRATES) [73] trial, evaluated septic shock and included patients with an endotoxin activity assay (EAA) ≥ 0.6 and a multiple organ dysfunction score (MODS) > 9 in the United States and Canada in 2018. The results showed a non-significant change in the 28-day mortality rates (44.5% in the PMX-DHP group vs. 43.9% in the conventional group, *p* = 0.92). Therefore, the SSCG 2021 [38] stated: “for adults with sepsis or septic shock, we suggest against using polymyxin B hemoperfusion. Weak recommendation, low quality of evidence.” However, the post hoc analysis of the EUPHRATES study excluding patients with an EAA > 0.9 showed a significant decrease in the 28-day mortality rates (26.1% in the PMX-DHP group vs. 36.8% in the conventional group, *p* = 0.047) [74]. Based on this, the safety and efficacy of polymyxin B hemoperfusion for endotoxemic septic shock in a randomized, open-label study (TIGRIS) is currently being conducted on patients with septic shock and EAA levels between 0.60 and 0.90 [75]. Moreover, in a meta-analysis of PMX-DHP for sepsis [76], PMX-DHP treatment reduced mortality in patients with sepsis. Specifically, the disease severity subgroup meta-analysis indicated a survival benefit related to the PMX-DHP treatment in intermediate- and high-risk groups, but not in the low-risk group. Thus, PMX-DHP should be considered for septic patients with high severity scores, such as the SOFA score and MODS.

## 12. Future Directions

BPT for patients with sepsis remains controversial because of the conflicting results obtained so far. However, these are likely attributed to the heterogeneity of patients included in the studies and/or differences in the timing, dose, or duration of therapy between the studies [77]. A more detailed understanding of the pathophysiology of sepsis and septic AKI and the development of appropriate biomarkers will help determine the phenotypes of sepsis that will benefit from BPT, as well as the optimal timing and type of therapy [78]. Furthermore, the need to focus on outcomes other than mortality, such as changes in organ dysfunction or hemodynamic status, in studies evaluating the appropriate strategies of BPT for sepsis has also been advocated [78]. Therefore, further studies focusing on the appropriate indications, optimal timing and selection of the membrane or cartridge, and optimal endpoints are needed in order to provide more evidence for the application of BPT for sepsis [77,78,79].

## 13. Conclusions

While the optimal method of KRT for AKI (renal indication) has been well discussed, that of BPT for sepsis (non-renal indication) remains controversial. Although the appropriate timing of CKRT initiation for AKI remains unclear, delayed initiation may increase the risk of death. On the other hand, excessive inflammation is an important factor in the development of sepsis, and BPT can reduce inflammatory mediators and improve hemodynamic instability. Given the pathophysiology of sepsis and the proven improvement in surrogate markers, BPT, especially that with cytokine-adsorbing hemofilters (AN69/AN69ST/oXiris, PMMA, and CytoSorb^®^), and PMX-DHP may decrease mortality in patients with sepsis. Further trials are needed in order to establish evidence of the effectiveness of BPT for sepsis.

## Figures and Tables

**Table 1:** Definitions of AKI.

	RIFLE [14]	AKIN [15]	KDIGO [16]
Diagnostic criteria	NA	Increase in serum creatinine level of ≥0.3 mg/dL or ≥50% within 48 h	Increase in serum creatinine level of ≥0.3 mg/dL within 48 h or ≥50% within 7 days
		OR	OR
		Urine output of <0.5 mL/kg/h for >6 h	Urine output of <0.5 mL/kg/h for > 6 h
Staging criteria			
Risk (RIFLE) or stage 1 (AKIN/KDIGO)	Increase in serum creatinine level to 1.5 times the baseline value	Increase in serum creatinine level of ≥ 0.3 mg/dL or to 150–200% of the baseline value	Increase in serum creatinine level of ≥ 0.3 mg/dL or 1.5–1.9 times the baseline value
	OR	OR	OR
	Urine output of < 0.5 mL/kg/h for 6–12 h	Urine output of < 0.5 mL/kg/h for 6–12 h	Urine output of < 0.5 mL/kg/h for 6–12 h
Injury (RIFLE) or stage 2 (AKIN/KDIGO)	Increase in serum creatinine level to 2 times the baseline value	Increase in serum creatinine level to 200–300% of the baseline value	Increase in serum creatinine level to 2.0–2.9 times the baseline value
	OR	OR	OR
	Urine output of < 0.5 mL/kg/h for 12–24 h	Urine output of < 0.5 mL/kg/h for 12–24 h	Urine output of < 0.5 mL/kg/h for 12–24 h
Failure (RIFLE) or stage 3 (AKIN/KDIGO)	Increase in serum creatinine level to 3 times the baseline value	Increase in serum creatinine level to > 300% of the baseline value	Increase in serum creatinine level to ≥ 3.0 times the baseline value
	OR	OR	OR
	Increase in serum creatinine level of 0.5 mg/dL to > 4.0 mg/dL	Increase in serum creatinine level by > 0.5 mg/dL to ≥ 4.0 mg/dL	Increase in serum creatinine level of ≥ 0.3 mg/dL to ≥ 4.0 mg/dL
	OR	OR	OR
	Urine output of < 0.3 mL/kg/h for > 24 h or anuria for > 12 h	Urine output of < 0.3 mL/kg/h for > 24 h or anuria for > 12 h	Urine output of < 0.3 mL/kg/h for ≥ 24 h or anuria for ≥ 12 h
	OR	OR	OR
	Initiation of kidney replacement therapy	Initiation of kidney replacement therapy	Initiation of kidney replacement therapy
Loss (RIFLE)	Need for kidney replacement therapy for > 4 weeks		
End stage (RIFLE)	Need for kidney replacement therapy for > 3 months		

AKI: acute kidney injury; RIFLE: risk, injury, failure loss, and end-stage; AKIN: acute kidney injury network; KDIGO: kidney disease improving global outcomes; NA: not applicable.

**Table 2:** Comparison of KRT initiation time for AKI.

Study	Design	Year	*n*	KRT Indication		Mortality	Difference	Consideration
ELAIN [39]	RCT	2016	231	Early	KDIGO stage 2 within 8 h	At 90 days	39.3% *p* = 0.03 54.7%	CKRT only
				Delayed	KDIGO stage 3 within < 12 h Absolute indication			
AKIKI [40]	RCT	2016	620	Early	KDIGO stage 3 within 6 h,	At 60 days	48.5% n.s 49.7%	CRBSI higher in early group than in the late group
				Delayed	hyperkalemia, metabolic acidosis, pulmonary edema, BUN level > 40 mg/dL, or oliguria for > 72 h			
IDEAL-ICU [41]	RCT	2018	488	Early	RIFLE-Failure within 12 h	At 90 days	58% n.s 54%	Hyperkalemia greater in the delayed group than in the non-delayed group Septic shock: 100%
				Delayed	Absence of kidney recovery after 48 h			
STARRT-AKI [42]	RCT	2020	2927	Early	KDIGO stage 2 or 3 within 12 h	At 90 days	43.9% n.s 43.7%	
				Delayed	K > 6.0 mmol/L, pH < 7.20, HCO3- level < 12 mmol/L, pulmonary edema with P/F < 200 for ≥ 72 h			
AKIKI-2 [43]	RCT	2021	278	Early	KDIGO stage 3 with oliguria > 72 h or BUN level 40–50 mmol/L within 12 h	At 60 days	44% n.s 55%	
				Delayed	BUN level > 140 mg/dL Absolute indication			

KRT: kidney replacement therapy; AKI: acute kidney injury; ELAIN: early vs. late initiation of renal replacement therapy in critically ill patients with acute kidney injury; RCT: randomized controlled trial; KDIGO: kidney disease improving global outcome; CKRT: continuous kidney replacement therapy; AKIKI: artificial kidney initiation in kidney injury; BUN: blood urea nitrogen; RIFLE: risk injury failure loss and end-stage kidney disease; n.s.: not significant; CRBSI: catheter-related bloodstream infection; IDEAL-ICU: initiation of dialysis early versus delay in the intensive care unit; STARRT-AKI: standard versus accelerated initiation of renal replacement therapy in acute kidney injury; P/F: partial pressure of arterial oxygen/fraction of inspired oxygen ratio.

## Data Availability

Not applicable.
